# Circular RNA-UBE2D2 accelerates the proliferation and metastasis of non-small cell lung cancer cells via modulating microRNA-376a-3p/Eukaryotic Translation Initiation Factor 4γ2 axis

**DOI:** 10.1080/21655979.2022.2027068

**Published:** 2022-02-23

**Authors:** JiChun Tong, JiaWei Lu, XiaoLiang Mao, Zheng Zhu, YeMin Wang, Ming Lou, Ke Zhang

**Affiliations:** Department of Cardiothoracic Surgery, Changzhou Second People’s Hospital, Changzhou City, Jiangsu Province, China

**Keywords:** Circular RNA-UBE2D2, non-small cell lung cancer, Eukaryotic Translation Initiation Factor 4γ2, proliferation

## Abstract

Non-small cell lung cancer (NSCLC) ranks first in the morbidity and mortality of malignant tumors in China. As reported, circular RNAs (circRNAs) are emerged in the progress of NSCLC. The study was to figure out the potential mechanism of circ-UBE2D2 in the progression of NSCLC. First, plasmid vectors intervening circ-UBE2D2, microRNA (miR)-376a-3p or Eukaryotic Translation Initiation Factor 4γ2 (EIF4G2) expression were transfected into NSCLC cells, and the expression of circ-UBE2D2, miR-376a-3p and EIF4G2 was detected by reverse transcription quantitative polymerase chain reaction or Western blot. Then, cell proliferation was detected by Cell counting kit-8 assay and plate cloning. Cell apoptosis was tested by flow cytometry. Plate scratches and Transwell were used to detect cell migration and invasion. Finally, the binding sites of circRNA UBE2D2, EIF4G2 and miR-376a-3p were verified by bioinformatics website starBase analysis and dual luciferase reporter gene assay. The results manifested the up-regulation of circ-UBE2D2 expression in NSCLC tissues and cells. Circ-UBE2D2 promoted the proliferation, migration and invasion, but repressed apoptosis of NSCLC cells. Interestingly, circ-UBE2D2 directly targeted miR-376a-3p and up-regulated miR-376a-3p restrained proliferation, migration and invasion, but accelerated apoptosis of NSCLC cells. More importantly, EIF4G2 was the target of miR-376a-3p, and overexpression of EIF4G2 reversed the effects of circ-UBE2D2 downregulation on proliferation, migration, invasion and apoptosis of NSCLC cells. These results suggest that circ-UBE2D2 promotes the proliferation, migration and invasion but restrains apoptosis of lung cancer cells by regulating miR-376a-3p/EIF4G2 axis.

## 1 Introduction

Lung cancer (LC) is a malignant tumor whose incidence is the vital cause of cancer-correlated deaths worldwide [1]. The most prevalent type is non-small cell lung cancer (NSCLC). NSCLC is regarded as an epithelial LC, taking up approximately 80–85% of all LCs [2]. Serious progress has been made in the prevention, diagnosis and treatment of NSCLC in recent decades [3,4]. Nevertheless, the 5-year overall survival rate (OS) of patients is still declined. Consequently, seeking for the effective and potential molecular targets was of great value for the treatment of NSCLC.

Non-coding RNAs (ncRNAs) are a class of RNA molecules that are not translated into proteins, exerting a crucial role in cancer diagnosis and treatment [5]. Abundant and functionally critical types of ncRNA cover microRNA (miRNA), circRNA, lncRNA and siRNA, among which circular RNA (circRNA), a specific type of ncRNA, is in any genomic region without usually coding for protein [6]. Several aberrant circRNAs are associated with the progression of LC [7–9]. Studies have clarified that circ-UBE2D2 is elevated in BC cell lines and tissues, predicts the poor prognosis [10], and strengthens Nolvadex resistance in breast cancer (BC) [11]. Nevertheless, the mechanism in which circ-UBE2D2 involves in the progression of NSCLC is yet unknown.

MicroRNAs (miRNAs) are associated with the occurrence, treatment and examination of NSCLC [12]. Foregoing studies have been elucidated that declined miR-376a-3p is a necessity for the occurrence and advancement of multiple cancers [13–16]. Eukaryotic Translation Initiation Factor 4γ2 (EIF4G2), a member of the eIFs family, is a translation activator during cellular stress [17–19]. Furthermore, aberrant EIF4G2 exerts a critical part in the progression of numerous cancers. For instance, repression of EIF4G2 is available to distinctively suppress the advancement of acute myeloid leukemia (AML) [20], diffuse large B-cell lymphoma (DLBCL) [21] and human osteosarcoma (HOS) [22]. Nevertheless, the function of EIF4G2 in NSCLC is poorly explored. It has been documented that the competitive endogenous RNA (ceRNA) networks are a crucial mechanism of circRNA in human diseases, targeting mRNA via sponge miRNA [23]. The identical miR-376a-3p complementary sites of circ-UBE2D2 with EIF4G2 were predicted, clarifying that circ-UBE2D2 was supposed to perform as the ceRNA of miR-376a-3p to accelerate EIF4G2. Circ-UBE2D2 in NSCLC tissues and cells was measured. Additionally, assessment of the functions of circ-UBE2D2 in cell advancement was performed, and the ceRNA network of circ-UBE2D2/miR-376a-3p/EIF4G2 in NSCLC cells was testified.

## 2 Methods

### 2.1 Clinical sample collection

A total of 48 NSCLC patients who did not adopt chemotherapy or radiotherapy were recruited. All participants signed an informed consent form to participate in the study and were authorized via the ethics committee of Changzhou Second People’s Hospital. During the operation, collection of the patient’s tumor tissue and para-cancerous tissues were conducted, and storing was performed until RNA separation.

### 2.2 Cell culture and transfection

Human lung cancer cell lines (A549, H1299, H1650) and BEAS-2B cells were performed (all the Chinese Academy of Sciences Type Culture Library Cell Bank, Shanghai, China), and culture of Dulbecco’s Modified Eagle Medium (Gibco, CarIsbad, CA) supplemented with 10% fetal bovine serum (FBS) (Invitrogen, CarIsbad, CA) and 1% penicillin-Streptomycin (Procell, Wuhan, China) was conducted. RiboBio (Guangzhou, China) was commissioned to construct si-NC, si-UBE2D2, oe-NC, oe-UBE2D2, mimic NC, miR-376a-3p mimic, si-UBE2D2 + inhibitor NC, si-UBE2D2 + miR-376a-3p inhibitor, si-UBE2D2 + pcDNA-NC, si-UBE2D2 + pcDNA-EIF4G2 oligonucleotide or vector. Transfection of these treated oligonucleotides or vectors was into A549 cells adopting Lipofectamine 2000 (Thermo Fisher, Wilmington, DE, USA) in line with the manufacturer’s instructions.

### 2.4 Reverse transcription quantitative polymerase chain reaction (RT-qPCR)

Separation of total RNA was from the cells or tissues adopting TRIzol Reagent (Invitrogen) in line with the manufacturer’s instructions, and reverse transcription of the RNA was into cDNA adopting PrimeScript RT Reagent Kit (Takara, Kusatsu, Japan). Analysis of RT-qPCR was done with SYBR Green PCR Kit (Takara, Japan). Adoption of U6 was to normalize the relative miR-376a-3p, and exertion of glyceraldehyde-3-phosphate dehydrogenase (GAPDH) was to normalize the relative abundance of circ-UBE2D2 and EIF4G2. Calculation of the relativity was done with the 2^−ΔΔCt^ method. The primer sequence is presented in Table 1.

**Table 1.** The primer sequences

Genes	Sequences
MiR-376a-3p	F: 5ʹ-CCCAGGAGGACTGAAGCAACAA-3’
	R: 5ʹ-GCTATCTCAGGGCTTGTTGCTTC-3’
U6	F: 5ʹ-CGCTTCGGCAGCACATATAC-3’
	R: 5ʹ-AAATATGGAACGCT-TCACGA-3’
Circ-UBE2D2	F: 5ʹ-AATGGCAGCATTTGTCTTGA-3’
	R: 5ʹ-GCCCCTGTGAGTAAGCTACG-3’
EIF4G2	F: 5ʹ-GGGTCATACTGCTGATTGTGGA-3’
	R: 5ʹ-GAATGTGGTGCTTTGCTTCTGT-3’
GAPDH	F: 5ʹ-ACGGCAAGTTCAACGGCACAG-3’
	R: 5ʹ-GACGCCAGTAGACTCCACGACA-3’

Note: F, forward; R, reverse.

### 2.4 Western blot

Extraction of the protein was adopting RNA immunoprecipitation analysis lysis buffer (Beyotime, Beijing, China), quantification was via the bicinchoninic acid method, and separation was via sodium dodecyl sulfate-polyacrylamide gel electrophoresis, and transfer was to polyvinylidene fluoride membrane. The primary antibody EIF4G2 (1:2000, 2182, Cell Signaling Technology), GAPDH (1:1000, 2118) and secondary horseradish peroxidase conjugated antibody (1:2000, 7074, Cell Signaling Technology) were adopted. Observation of the protein bands was done with electrochemiluminescence.

### 2.5 Cell counting kit (CCK-8)

Collection and storing of the cells were in a 96-well plate with 2.5 × 10^3^ cells/wells. Addition of 10 μL CCK-8 assay reagent (Dojindo, Tokyo, Japan) was performed after 24, 48, 72 h. After 2 h, accretion of Dimethyl Sulfoxide was at a final concentration of 0.1%. Subsequently, examination of the absorbance of the solution at 450 nm was done with a microplate reader.

### 2.6 Plate cloning

Seeding of approximately 500 cells/well was in a 6-well plate (BD Falcon, Shanghai, China). Culture was for 14 d until the colony was huge enough to be clearly distinguished. Staining of the colonies was with a solution covering 0.5% crystal violet and 25% methanol, and the number of colonies was counted.

### 2.7 Flow cytometry

Examination of the cell apoptosis was with the Annexin V-fluoresceinisothiocyanat (FITC)/propidium iodide (PI) Apoptosis Kit (Keygen, Nanjing, China) in line with the manufacturer’s instructions. After incubation of the A549 cells for 72 h, preparation of the cell suspension was with 0.125% trypsin, and centrifugation was performed for 5 min. Then, resuspension of the cells was in binding buffer (10 mM 4-(2-hydroxyethyl)-1-piperazineëthanesulfonic acid, pH 7.4, 140 mM NaCl and 2.5 mM CaCl2, KeyGEN, Nanjing, China) at a concentration of 1 × 10^6^ cells/mL. Subsequently, staining was done with Annexin V-FITC and PI, and analysis was conducted.

### 2.8 Plate scratches

When the cell fusion was 90%, a vertical wound was formed in each well with a 20 μL pipette tip, and images were taken at 0 and 24 h. The gap distance of the original wound was set as 100%. Random selection of three areas was to measure the gap distance after 24 h, which was quantified and normalized in line with the gap distance of the original wound. Calculation of the migration rate was via measuring the cell migration length and compared with the control. Migration rate = (migration distance of cells in the treatment/migration distance of cells in the control) × 100%.

### 2.9 Transwell

Transwell invasion test was performed adopting a transwell chamber (Keygen, Nanjing, China) involving matrix gel with 8 μm pore size. The cells are digested and counted. Placing of 1 × 10^6^ cells was in the upper chamber with 100 μL FBS free medium, and the lower chamber was covered with 500 μL medium containing 10% FBS as a chemical attractant. After incubation in a wet incubator for 24 h, removal of non-migrating cells was on the upper membrane surface, fixation of the cells on the lower membrane surface was with 4% polyoxymethylene (Sigma, MO, USA), and staining was with 0.1% crystal violet (Sigma, MO, USA). The cells were counted and the images were recorded via shooting five random fields at 400 × magnification under a microscope (BX53, Olympus, Tokyo, Japan).

### 2.10 The luciferase reporter experiment

Cloning of the binding and mutation sequences of miR-376a-3p in circ-UBE2D2 or EIF4G2 was into pmiR-RB-Report (Promega, Shanghai, China). After that, co-transfection of the pmiR-RB-Report luciferase vector covering wild type (WT) or mutant (MUT) circ-UBE2D2 or EIF4G2 and miR-376a-3p mimic or mimic NC was to A549 cells adopting LipofectamineTM 3000 (Invitrogen). Ultimately, analysis of the luciferase activity was done with a dual luciferase detection kit (Promega).

### 2.10 Data statistics

All data were processed via SPSS 21.0 statistical software (SPSS, Inc, Chicago, IL, USA). Representation of measurement data was in the form of mean ± standard deviation (SD), the comparison between the two of measurement data subjecting to normal distribution was adopting t test, and the comparison between the multiple was via one-way analysis of variance (ANOVA) and Tukey’s post-hoc test. Comparison of the data between groups at different time points was performed adopting repeated measures ANOVA, and Bonferroni post-hoc test. Analysis of the correlation of circ-UBE2D2, miR-376a-3p and EIF4G2 in clinical samples was via Pearson. *P* < 0.05 was accepted as indicative of distinct differences.

## 3 Results

### 3.1 Circ-UBE2D2 is elevated in NSCLC tissues and cells

**Figure 1.**
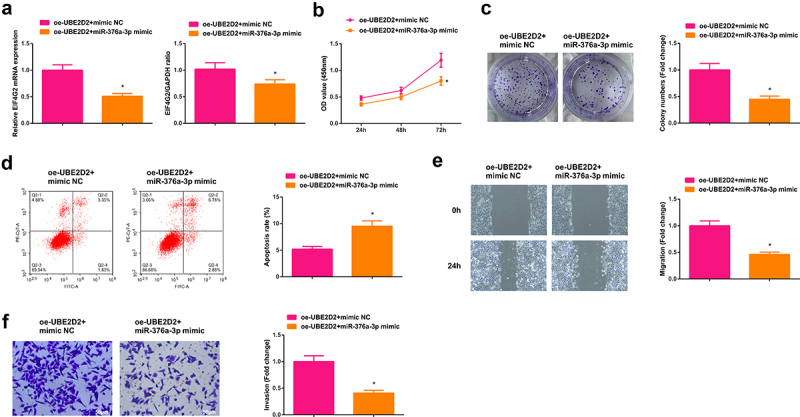
Circ-UBE2D2 is elevated in NSCLC tissues and cells.

Collection of 48 pairs of tumors and para-cancerous normal tissues with NSCLC patients was used to explore the aberrant circRNA in NSCLC. Circ-UBE2D2 was distinctively elevated in NSCLC tissues, as proved in Figure 1(a). Analysis of the clinical information table was performed (Table 2), which clarified that circ-UBE2D2 was correlated with tumor node metastasis (TNM) staging and lymph node metastasis, and elevated circ-UBE2D2 predicted a poor prognosis (HR = 1.80; *P* < 0.05; 95% CI, 0.32–1.41) (Figure 1(b)). Additionally, circ-UBE2D2 was elevated in NSCLC cell lines (A549, H1299, H1650), among of which A549 cells were the most augmented (Figure 1(c); therefore, selection of A549 cells was for subsequent experiments. In short, circ-UBE2D2 was elevated in NSCLC.

**Table 2.** Correlation is of miR-181b-5p gene and clinical features in patients with NSCLC

Characteristics	Number	Circ-UBE2D2		*P*
		The elevated (n = 24)	The declined (n = 24)	
Age				0.770
Less than 60	28	15	13	
60 or more	20	9	11	
Gender				0.773
Male	24	13	11	
Female	24	11	13	
Smoking Status				0.341
Ever	34	15	19	
Never	14	9	5	
Histological classification				1.000
SCC (Squamous cell carcinoma)	19	10	9	
AD (adenocarcinoma or other)	29	14	15	
Tumor Size				0.359
3 cm or less	16	6	10	
More than 3 cm	32	18	14	
TNM Stage				0.002
I + II	17	3	14	
III + IV	31	21	10	
Lymphatic metastasis				Less than 0.001
Yes	35	12	23	
No	13	12	1

A: Test of circ-UBE2D2 in LC tissues and para-cancerous tissues was via RT-qPCR (N = 48); B: Survival prognostic analysis; C: Examination of circ-UBE2D2 in LC cell lines was via RT-qPCR. The data in the figure were all measurement data, and the values were expressed as mean ± SD, *P* < 0.05.

### 3.2 Silence of circ-UBE2D2 suppresses the advancement of NSCLC cells

**Figure 2.**
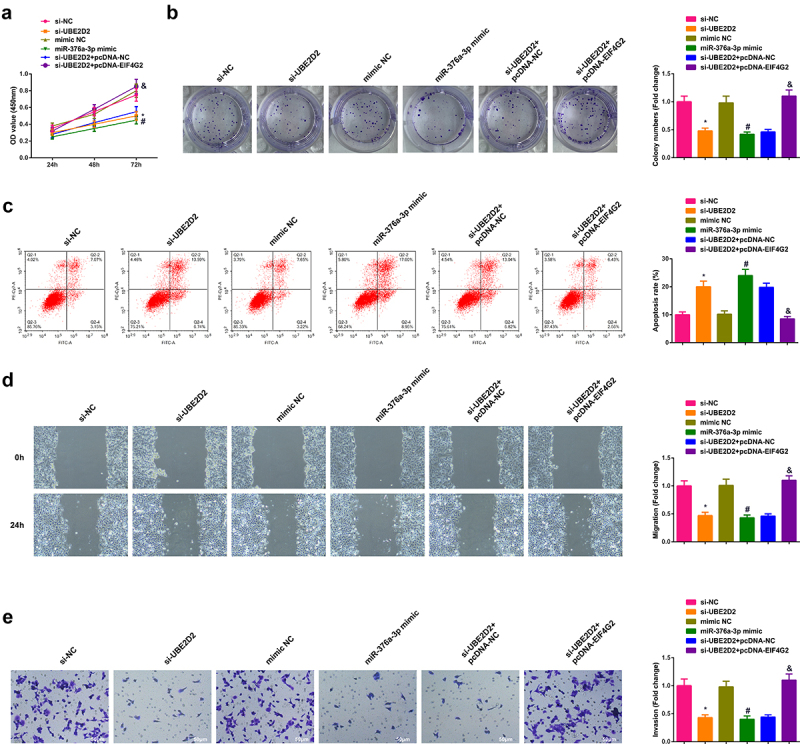
Silence of circ-UBE2D2 restrains the proliferation, migration and invasion of NSCLC cells.

Transient transfection of si-NC, si-UBE2D2, oe-NC, and oe-UBE2D2 was into A549 cells to figure out the action of circ-UBE2D2 in the progression of NSCLC, and the successful transfection was via PCR (Figure 2(a)). Cell proliferation was detected by CCK-8 and plate cloning, and the results manifested that the proliferation of A549 cells was reduced after UBE2D2 was down-regulated (Figure 2(b,c)). Cell apoptosis was detected by flow cytometry, and it was found that apoptosis of A549 cells was elevated after UBE2D2 was down-regulated (Figure 2(d)). The migration and invasion of A549 cells were detected by plate scratches and Transwell assay, and the results clarified that down-regulation of circ-UBE2D2 could repress the migration and invasion of A549 cells (Figure 2(e,f)). In contrast, up-regulation of circ-UBE2D2 showed the opposite result (Figure 2(b-f)). In brief, silence of circ-UBE2D2 repressed the advancement of NSCLC cells.

A: Test of circ-UBE2D2 in A549 cells of each group was via RT-qPCR; B-C: Examination of the cell proliferation was via CCK-8 and plate cloning; D: Test of the cell apoptosis was via flow cytometry; E: Detection of the cell migration was via plate scratch; F: Examination of the cell invasion was via Transwell. The data in the figure were all measurement data, and the values were expressed via the mean ± SD, * *P*< 0.05 versus the si-NC; #*P* < 0.05 versus the oe-NC.

### 3.3 Circ-UBE2D2 directly combines with miR-376a-3p and modulates miR-376a-3p

**Figure 3.**
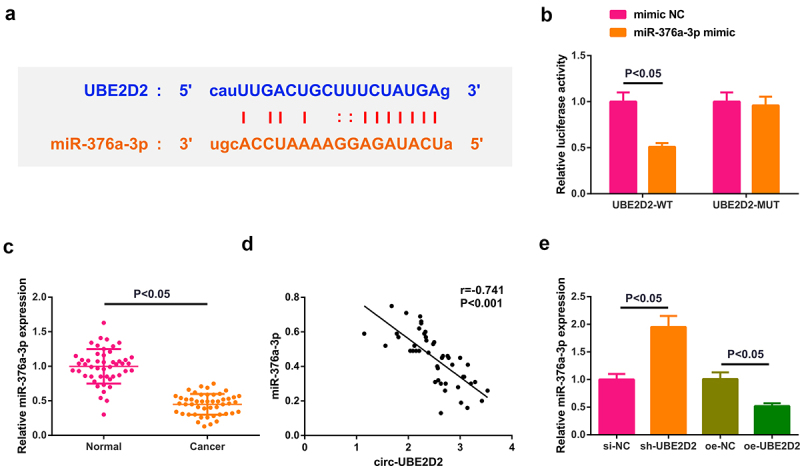
Circ-UBE2D2 directly combines with miR-376a-3p and controls miR-376a-3p.

Detailed analysis of miRNAs that were supposed to be combined with circ-UBE2D2 was performed. Test of the potential binding sites of circ-UBE2D2 with miR-376a-3p was conducted (Figure 3(a)), which elucidated that miR-376a-3p mimic declined the luciferase activity of the UBE2D2-WT reporter plasmid (Figure 3(b)). Additionally, it was also found that miR-376a-3p expression was down-regulated in clinical samples, and circ-UBE2D2 was negatively correlated with miR-376a-3p (Figure 3(c,d)). Ultimately, detection of miR-376a-3p in A549 cells of transfecting with UBE2D2 was performed, which elucidated that miR-376a-3p was declined after elevation of UBE2D2, while miR-376a-3p was augmented after silence of UBE2D2 (Figure 3(e)). In general, circ-UBE2D2 directly conjugated with miR-376a-3p and modulated miR-376a-3p.

A: Binding sites of circ-UBE2D2 and miR-376a-3p; B: Verification of the targeting of circ-UBE2D2 with miR-376a-3p was with the luciferase digestion report experiment; C: Detection of miR-545-5p in LC tissue and para-cancerous was via RT-qPCR (N = 48); D: The correlation was of circ-UBE2D2 with miR-376a-3p; E: Detection of miR-376a-3p in A549 cells of each group was via RT-qPCR. The data in the figure were all measurement data, and the values were expressed as mean ± SD.

### 3.4 Augmentation of miR-376a-3p represses the advancement of NSCLC cells

**Figure 4.**
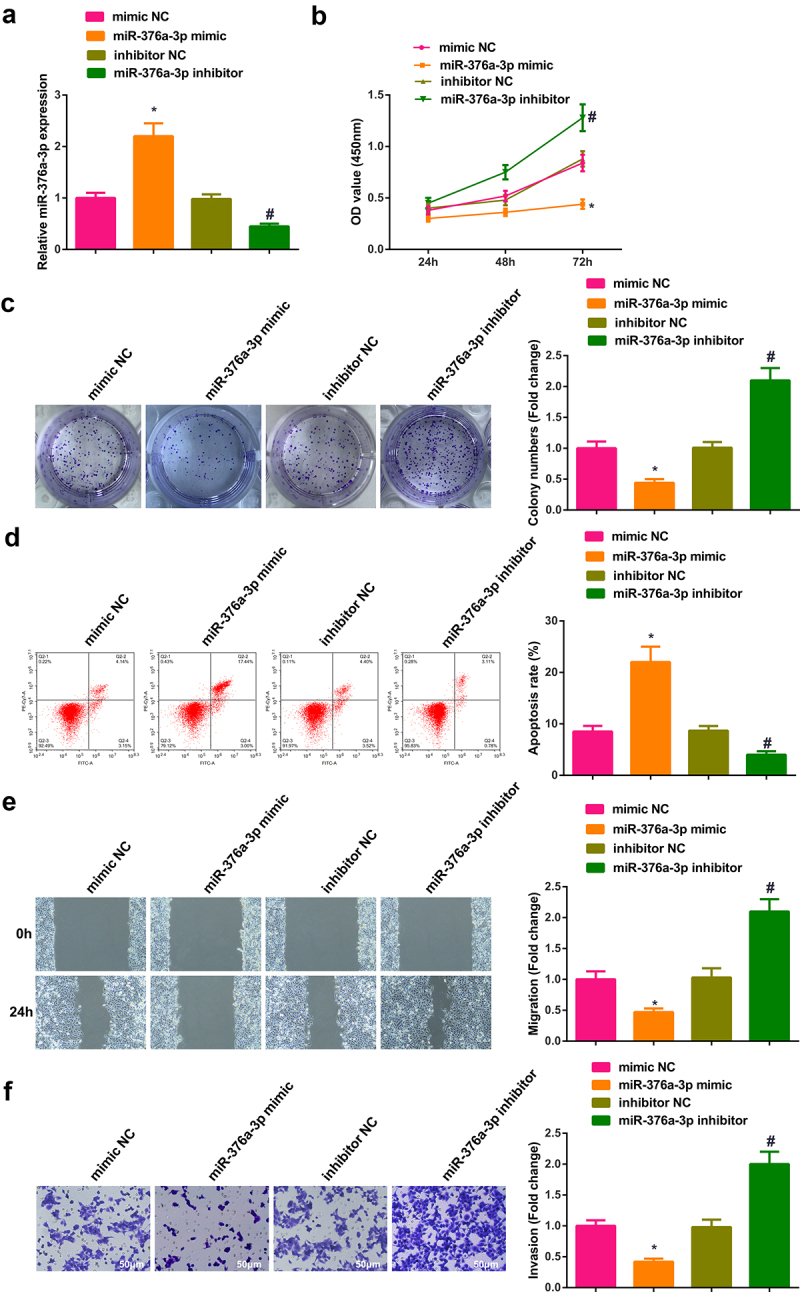
Augmentation of miR-376a-3p represses the advancement of NSCLC cells.

Transient transfection of mimic NC, miR-376a-3p mimic, inhibitor NC and miR-376a-3p inhibitor was into A549 cells to figure out the action of miR-376a-3p in the progression of NSCLC, and the successful transfection was via PCR (Figure 4(a)). Cell proliferation was detected by CCK-8 and plate cloning; cell apoptosis was detected by flow cytometry, and cell migration and invasion abilities were detected by plate scratches and Transwell method. The experimental results manifested that after up-regulation of miR-376a-3p, the proliferation, migration and invasion abilities of A549 cells were weakened, but cell apoptosis was elevated. The results of down-regulating miR-376a-3p were all opposite (Figure 4(b-f)). In general, augmentation of miR-376a-3p restrained the advancement of NSCLC cells.

A: Test of miR-376a-3p in A549 cells of each group was via RT-qPCR; B-C: Examination of the cell proliferation was via CCK-8 and plate cloning; D: Test of the cell apoptosis was via flow cytometry; E: Detection of the cell migration was via plate scratch; F: Examination of the cell invasion was via Transwell. The data in the figure were all measurement data, and representation of the values was via the mean ± SD, * *P*< 0.05 versus mimic NC; #*P* < 0.05 versus the inhibitor NC.

### 3.5 Circ-UBE2D2 elevates EIF4G2 via miR-376a-3p

**Figure 5.**
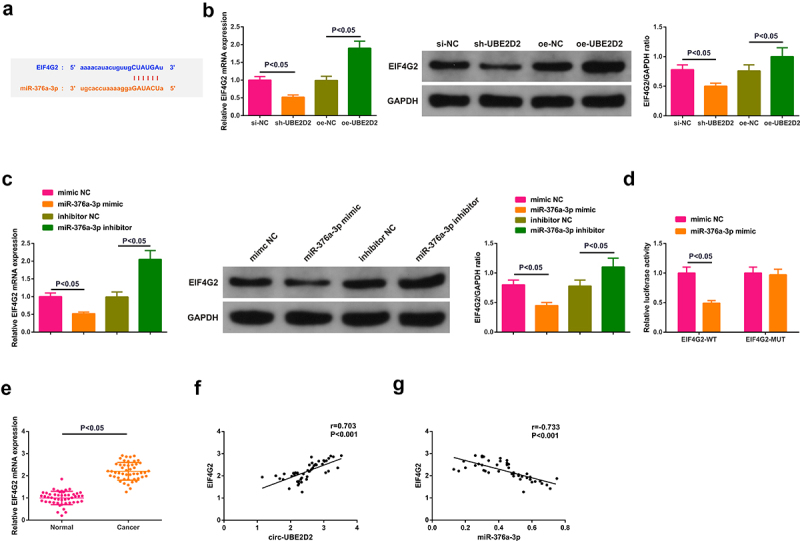
Circ-UBE2D2 elevates EIF4G2 via miR-376a-3p.

The potential target gene EIF4G2 of miR-376a-3p was predicted (Figure 5(a)). Test of EIF4G2 in A549 cells of interfering circ-UBE2D2 andmiR-376a-3p was performed to determine whether circ-UBE2D2 or miR-376a-3p was able to modulate EIF4G2. The results manifested that elevation of circ-UBE2D2 distinctively elevated EIF4G2, while knockdown declined EIF4G2 (Figure 5(b)). Augmentation of miR-376a-3p also distinctively declined EIF4G2, and silence of miR-376a-3p critically elevated EIF4G2 (Figure 5(c)). Construction of an untranslated region (UTR) vector involving miR-376a-3p binding site was tested with the luciferase reporter gene detection to verify the interaction of miR-376a-3p with EIF4G2 wild-type (EIF4G2-WT) and mutant (EIF4G2-MUT). The results clarified that the luciferase activity was critically declined after co-transfection of EIF4G2-WT vector and miR-376a-3p mimic (Figure 5(d)), manifesting that miR-376a-3p was available to directly combine with EIF4G2. Additionally, EIF4G2 was elevated in clinical samples, and EIF4G2 was positively correlated with circ-UBE2D2, while EIF4G2 was negatively associated with miR-376a-3p (Figure 5(e-g)). In brief, EIF4G2 was positively modulated via circ-UBE2D2 and negatively modulated via miR-376a-3p. In addition, it was also found that up-regulation of miR-376a-3p could reverse the positive regulation of up-regulated circ-UBE2D2 on EIF4G2 expression, and also turned around the promoting effect of elevated circ-UBE2D2 on NSCLC progression (Attached Figure 1(a-f)), which further proved the conclusion.

A: Binding sites of miR-376a-3p and circ-UBE2D2; B/C: Test of miR-376a-3p in A549 cells of each group was via RT-qPCR; D: Verification of the targeting of circ-UBE2D2 with miR-376a-3p was via the dual luciferase digestion report experiment; E: Examination of EIF4G2 in LC tissue and para-cancerous tissues was via RT-qPCR (N = 48); F: The correlation was of circ-UBE2D2 with EIF4G2; G: The association was of miR-376a-3p with EIF4G2. The data in the figure were all measurement data, and representation of the values was as mean ± SD.

### 3.6 Circ-UBE2D2 accelerates the progression of NSCLC via modulating miR-376a-3p/EIF4G2 axis

**Figure 6.**
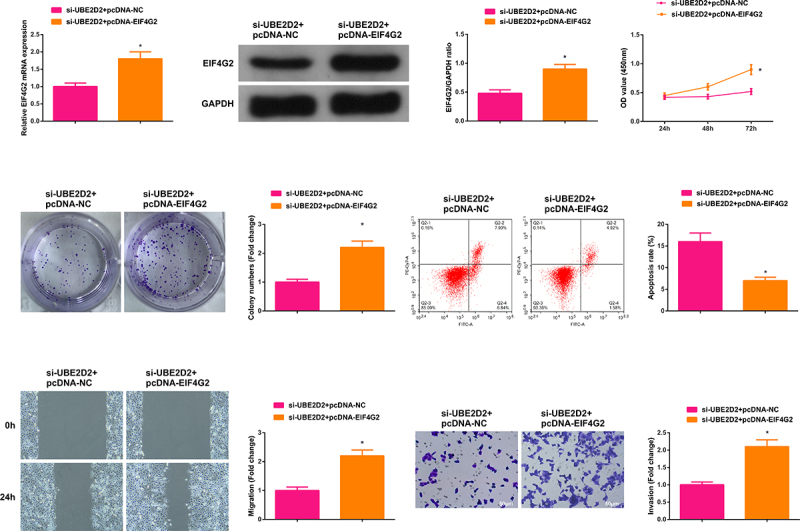
Circ-UBE2D2 facilitates the progression of NSCLC via modulating miR-376a-3p/EIF4G2 axis.

Transient transfection of si-UBE2D2 + pcDNA-NC and si-UBE2D2 + pcDNA-EIF4G2 was into A549 cells to figure out the function of circ-UBE2D2/miR-376a-3p/EIF4G2 axis in the progression of NSCLC, and the successful transfection was via PCR and WB (Figure 6(a)). Examination of the cell proliferation was conducted; Test of the cell advancement was performed. The results elucidated that the elevation of EIF4G2 turned around the repression of silence of circ-UBE2D2 on the proliferation of A549 cells (Figure 6(b-e)). Furthermore, the same experiment was performed in H1650 cells and the results were consistent with those in A549 cells (Attached Figure 2(a-e)). In brief, circ-UBE2D2 boosted the proliferation of NSCLC via modulating miR-376a-3p/EIF4G2 axis.

A: Test of EIF4G2 in A549 cells of each group was via RT-qPCR and Western blot; B-C: Examination of the cell proliferation was via CCK-8 and plate cloning; D: Test of the cell apoptosis was via flow cytometry; E: Detection of the cell migration was via plate scratch; F: Examination of the cell invasion was via Transwell. The data in the figure were all measurement data, and representation of the values was as mean ± SD, *P < 0.05 versus the si-UBE2D2 + pcDNA-NC.

## 4 Discussion

Clinically, only a minority of patients with NSCLC have been diagnosed early and treated with surgical resection. Diagnosis of over 60% NSCLC patients is with advanced or metastatic tumors [24]. Consequently, it is a necessity to seek for novel biomarkers and therapeutic targets for the effective diagnosis and treatment of NSCLC. Recently, circRNA is not regarded as a random product in the RNA shearing any more whose biological value and function in malignant tumors have been drawn more and more attention. Foregoing reports have manifested that circ_0001073 restrains the survival and invasion of LC cells [25]; Circ-CCS is elevated in LC, being available to predict the poor prognosis [26]. Circ-UBE2D2 is elevated in BC cell lines and tissues, predicts the poor prognosis [10], and strengthen Nolvadex resistance in BC [11]. In the study, circ-UBE2D2 in NSCLC tissues and cells was critically augmented. Besides, circ-UBE2D2 is able to boost the proliferation of NSCLC cells.

Endogenous circRNA is available to perform as microRNA sponge to suppress its function, and several studies have associated miRNA sponges with human diseases involving cancer [27]. Foregoing studies have manifested that circ_0020123, as ceRNA of miR-488-3p, modulates ADAM9 to boost the progression of NSCLC [28]. Decline of miR-376a-3p is a necessity for the occurrence and advancement of multiple cancers [13–16]. Nevertheless, the potential association of miR-376a-3p with circ-UBE2D2 has not been figured out. In the study, circ-UBE2D2 directly targeted miR-376a-3p, and elevation of miR-376a-3p was able to restrain the proliferation of NSCLC cells. These data offered clues to treatment strategies for NSCLC.

MiR-376a-3p was available to directly target EIF4G2 in NSCLC cells. Multiple studies have elucidated that EIF4G2-dependent mRNAs are specifically emerging in cancer-facilitating activities like cell proliferation, anti-apoptosis, tumor invasion, metastasis and angiogenesis [17,19,24–33]. Thus, EIF4G2 was associated with the development and treatment of tumor. Silence of EIF4G2 is available to stimulate ovarian cancer (OC) patients to regain chemosensitivity to paclitaxel [34], and strengthen the sensitivity of cisplatin chemotherapy in NSCLC [35]. The data of this study clarified that EIF4G2 in NSCLC was augmented. Additionally, elevation of EIF4G2 turned around the influence of silence of circ-UBE2D2 on the proliferation of NSCLC, clarifying that circ-UBE2D2 boosted the progression of NSCLC via the miR-376a-3p/EIF4G2 axis. However, there are still some limitations in this study. First, the function of circ-UBE2D2/miR-376a-3p/EIF4G2 axis *in vivo* was not explored in this study. The LC cell lines with stable intervention of circ-UBE2D2, miR-376a-3p, and EIF4G2 expression are constructing to investigate the effects of circ-UBE2D2/miR-376a-3p/EIF4G2 axis on tumor growth *in vivo*.

## 5. Conclusion

In brief, the mechanism in which circ-UBE2D2 directly combines with miR-376a-3p to augment EIF4G2 and facilitate the proliferation of NSCLC cells is uncovered. This work not only offers a theoretical basis for a better comprehension of the mechanism of circ-UBE2D2, but circ-UBE2D2 or miR-376a-3p is supposed to be considered as biomarkers of NSCLC. This study provides a new target for the treatment of NSCLC, and it is recommended that more studies are carried out to verify the function of circ-UBE2D2/miR-376a-3p/EIF4G2 axis in the progression of NSCLC.
